# Editorial: Organotins as a Complete Physiologic and Endocrine Disruptor: Role of Disease Development

**DOI:** 10.3389/fendo.2019.00799

**Published:** 2019-11-26

**Authors:** Jones Bernardes Graceli

**Affiliations:** Department of Morphology, Federal University of Espírito Santo, Vitória, Brazil

**Keywords:** organotins, endocrine disrupting chemicals, physiology, dysfunction, disease

The metal tin and its alloys have a historically important role for humanity. Studies have reported the existence of organotin (OT) since 1853, but it did not become important in industrial use until the 1940–1960s (Marques et al.). OTs are synthetic chemical tetravalent derivatives of tin (IV) with a general formula of R(4-n) SnXn, where R represents organic substituents and X can be a halide, anion, or an organic group linked covalently through a heteroatom (O, N, S, Cl, etc.) (Nunes-Silva et al.). Mono-, di-, tri-, and tetra-OT have many industrial applications, including as PVC catalysts and broad-spectrum biocides, as well as in antifouling paints for marine ships (Nunes-Silva et al.). As a consequence, the main inputs of tri-OT tributyltin (TBT) into the environment are through contamination of water and sediments by improper disposal of antifouling products. TBT has a degradation half-life of days to months in water and up to several years in sediment. TBT is accumulative in different organisms along of the food chain (Fernandez). Thus, aquatic organisms can be exposed by a contaminated habitat (water and sediment) and/or ingestion of contaminated food. Terrestrial organisms may also be exposed via OT- and TBT-contaminated sediments and by the intake of contaminated food or water. Thus, for the majority people, the main route of OT exposure is intake by consumption of contaminated water and foods, as for example, marine foods (Marques et al.; Nunes-Silva et al.; Fernandez).

Through previous studies, we have learned that the use of TBT as the active component in the marine antifouling ship paints has increased because of its particularly potent algicide/molluscicide effects (Fernandez; Vogt et al.). For example, OT exposure (mainly as TBT) can lead to imposex development, the abnormal induction of male sex features in female gastropod mollusks, representing one the clearest examples of environmental endocrine disruption chemical (EDC) action (Marques et al.; Nunes-Silva et al.; Fernandez; Vogt et al.). In addition, another study has shown that TBT exposure can also induce masculinization in fish species (Berto-Júnior et al.). Widespread environmental contamination of marine ecosystems with TBT began in the 1960s, leading to several adverse effects in numerous organisms. Therefore, for this reason, its use in antifouling ship paints was prohibited by the International Marine Organization (IMO) in 2008 (Fernandez; Vogt et al.). However, beyond its continued utilization in industrial and other processes, it is possible that TBT is still employed in some parts of the world, particularly in countries that are not included in the Antifouling Systems (AFS) convention and/or have poor environmental monitoring/supervision (Fernandez; Barbosa et al.). Unfortunately, previous investigation has confirmed that recreational vessels sampled from north European countries contain high TBT levels in their paints and may be a source of it into the environment (Fernandez; Barbosa et al.). Other important recent studies have shown a higher level of OT pollution in commercial and wild oysters from Asia (Fernandez; Barbosa et al.).

OTs are a diverse organometallic group of widely distributed environmental xenobiotics, and, today, more than 800 OTs are known (Marques et al.; Nunes-Silva et al.; Fernandez; Vogt et al.; Berto-Júnior et al.; Barbosa et al.). OTs have complex toxicological effects on both invertebrate and vertebrate endocrine systems (Fernandez; Vogt et al.; Barbosa et al.; de Araújo et al.). The story of OTs, as well as that of TBT, is far from reaching an end; in fact, the discovery of its new potential EDC actions has placed it again at the forefront of scientific research. In the USA, more than 80,000 chemicals are registered with the Environmental Protection Agency (EPA), some of which are known or potential EDCs. About 1,000 synthesized chemicals are considered to be EDCs, defined as “exogenous chemical[s], or mixtures of chemicals, that interfere with any aspect of hormone action, as organotin chemicals” (de Araújo et al.). A number of issues have proven to be key to a full understanding of the toxicological mechanisms of action and consequences of exposure to OT as an EDC, for example, age at exposure, latency from exposure, the importance of mixtures with other EDCs, non-traditional dose-response dynamics, and transgenerational, epigenetic effects (Marques et al.; Nunes-Silva et al.; Fernandez; Vogt et al.; Berto-Júnior et al.; Barbosa et al.; de Araújo et al.).

Several *in vitro* and *in vivo* studies on gastropods, crustaceans, amphibians, fish, rodents, and humans demonstrated that TBT is able to interfere with many physiologic processes, thereby inducing complex toxic effects (Marques et al.; Nunes-Silva et al.; Fernandez; Vogt et al.; Berto-Júnior et al.; Barbosa et al.; de Araújo et al.). A wide range of detrimental responses are observed in mollusks, crustaceous, cephalopods, fish, and amphibians exposed to low levels of TBT (0.1–100 ng/L), such as imposex, apoptosis, irregular metamorphosis, and other important abnormalities (Fernandez; Berto-Júnior et al.; de Araújo et al.). Additionally, OT may accumulate in birds and sea mammals, leading to reproductive and metabolic dysfunctions (Fernandez; de Araújo et al.). In rodent models, toxicological studies have shown reproductive, cardiovascular, renal, respiratory, neural, and other abnormalities with different TBT doses (100 ng−100 mg/Kg) (Marques et al.; Nunes-Silva et al.; Barbosa et al.; de Araújo et al.; Ronconi et al.; Ferraz da Silva et al.). Therefore, data in different animal models demonstrate the deleterious effects of TBT exposure on multiple organ and species systems.

TBT is an obesogenic chemical (EDC-subclass) thought to induce obesity and other metabolic abnormalities by increasing the number and/or size of fat cells and/or altering the mechanisms through which the body regulates appetite and satiety (Berto-Júnior et al.). Therefore, obesogen chemicals display the potential to disrupt multiple metabolic pathways in the developing organism, which might result in permanent changes in adult physiology, in various experimental species models (Berto-Júnior et al.; de Araújo et al.). Several TBT obesogenic effects are mediated by PPAR-γ signaling, which acts as a key regulator of adipocyte differentiation and as a transcriptional regulator and/or effector of target genes, such as C/EBP (CCAAT/enhancer-binding proteins), AFABP (adipocyte-specific fatty acid-binding protein), and FATP (fatty acid transport protein) (Berto-Júnior et al.; de Araújo et al.).

TBT is able to impair different physiology functions as result of an increase in oxidative stress processes (Marques et al.; Ronconi et al.). Rodent studies reported that TBT exposure (100 ng/kg/day) led to reproductive tract, neuronal, renal, and cardiovascular oxidative stress (Marques et al.; de Araújo et al.; Ronconi et al.; Ferraz da Silva et al.). Additionally, TBT exposure (0, 1, 10, and 100 ng L^−1^) played a key role as an inducer of oxidative stress and a positive modulator of pro-inflammatory cytokines in a zebrafish model (Berto-Júnior et al.).

This Research Topic brings together nine review papers on the different and complex toxicological role of organotin in the environment, in wild species, such as crustaceans, gastropods, amphibians, and fish, and in rodent and human experimental models. Understanding the interplay between organotin, as well as TBT exposure from different sources, and physiological abnormalities is highly relevant for wildlife and human health. Evidently, investigation in this field is advancing at a rapid pace. The articles in this Research Topic highlight novel findings and unanswered questions for future investigation.

## Author Contributions

The author confirms being the sole contributor of this work and has approved it for publication.

### Conflict of Interest

The author declares that the research was conducted in the absence of any commercial or financial relationships that could be construed as a potential conflict of interest.

